# Chemical Study and Comparison of the Biological Activities of the Essential Oils of *Helichrysum petiolare*, *H. cymosum*, and *H. odoratissimum*

**DOI:** 10.3390/plants11192606

**Published:** 2022-10-03

**Authors:** Selena O. Adewinogo, Rajan Sharma, Charlene W. J. Africa, Jeanine L. Marnewick, Ahmed A. Hussein

**Affiliations:** 1 Chemistry Department, Cape Peninsula University of Technology, Bellville Campus, Symphony Road, Bellville 7535, South Africa; 2 Medical Biosciences, University of the Western Cape, Bellville 7535, South Africa; 3 Applied Microbial and Health Biotechnology Institute, Cape Peninsula University of Technology, Symphony Rd., Bellville 7535, South Africa

**Keywords:** essential oils, *Helichrysum*, *H. petiolare*, *H. odoratissimum*, *H. cymosum*, antioxidant, antibacterial, tyrosinase inhibition, sun protection factor

## Abstract

*Helichrysum* species are prominent South African medicinal plants. From the essential oils (EOs) of three *Helichrysum* species, *H*. *petiolare*, *H*. *odoratissimum*, and *H*. *cymosum*, sixty-three constituent components were identified, with hydrocarbons and oxygenated monoterpenes and sesquiterpenes as major components. The compounds were analyzed by gas chromatography-mass spectrometry (GC-MS) and nuclear magnetic resonance (NMR) spectroscopy. In *H*. *petiolare* EO, the major components were faurinone (20.66%) and (E)-β-ocimene (17.21%). Faurinone was isolated from this EO for the first time. In *H*. *odoratissimum*, 1,8-cineole (17.44%) and α-pinene, and γ-curcumene (15.76%) were the major components whereas, in *H*. *cymosum*, α-pinene (29.82%) and (*E*)-caryophyllene (19.20%) were the major components. In the antibacterial activity study, the EOs were tested against *Staphylococcus aureus*, *Escherichia coli*, and *Pseudomonas aeruginosa*. The EOs were found to possess low antibacterial, anti-tyrosinase, and photoprotection activities and moderate antioxidant capacities, thus establishing these *Helichrysum* EOs as valuable antioxidant agents.

## 1. Introduction

*Helichrysum* Mill. is a large genus comprising over 600 species spread throughout Africa, Europe, North America, and Australia. Out of these, nearly 244–250 *Helichrysum* species occur in Southern Africa (including Namibia) with extensively varied morphologies [1,2]. *Helichrysum* species are popular materials in the traditional medicines of Europe, Asia, and Africa, where their herbal teas are used to treat fever, cough respiratory problems, digestive disorders, skin inflammation, and wounds [3,4,5].

Essential oils (EOs) and their volatile constituents have been important materials for preventing and treating human diseases for a long time [6]. *Helichrysum* EOs are well studied in the literature [7,8,9,10] and show promising biological potencies. *H. italicum* EO, also called “immortelle”, is already a renowned ingredient of cosmetics. It is said to promote blood flow in the skin, regenerate it, and help attenuate signs of aging such as fine lines and wrinkles [5].

As the ethnomedicinal records propose *Helichrysum* species as a skin remedy in the quest to explore the South African flora for novel cosmeceutical ingredients, *H. petiolare*, *H. odoratissimum*, and *H. cymosum* EOs were selected for phytochemical investigation. Table 1 summarizes the studies related to the EO’s of these *Helichrysum* species with regard to chemical composition and biological activity.

## 2. Results and Discussion

### 2.1. Yield and Chemical Composition of the Helichrysum Essential Oils

The hydrodistilled plant materials of *H*. *petiolare* and *H*. *cymosum* were isolated as pale green and clear essential oils, respectively. *H*. *petiolare* had a higher essential oil yield of 0.25% (*v/w*) against 0.15% (*v/w*) for *H*. *cymosum*.

The *Helichrysum* essential oils were found to collectively contain high amounts of α-pinene up to 29.82% as in *H*. *cymosum* EO and 1,8-cineole up to 17.44% as in *H*. *odoratissimum* EO, as shown in Table 2. These compounds have previously been reported as major constituents in the GC-MS analyses of these *Helichrysum* species. α-Pinene has been reported in African *H. odoratissimum* EOs in amounts as high as 43.4% and 40.6–47.1% [19,20]. Lourens et al. [21] have reported 1,8-cineole (22.4%) as the major compound in *H*. *petiolare* from the plant collected in Pretoria. Reddy [12] found 1,8-cineole as the major compound in *H*. *cymosum* EO as 20.4–34.6%. In *H*. *odoratissimum* EO, 1,8-cineole compound has been found between 6.56 and 17.1% [5,12,15,16].

*H*. *odoratissimum* and *H*. *cymosum* EOs both featured a high content of (*E*)-caryophyllene at 7.30% and 19.20%, respectively, as was previously identified in previous reports by Reddy [12] (9.3–25.2%), and Bougatsos et al. [8] (27.04%). (*E*)-β-ocimene found in high content in *H*. *petiolare* EO (17.21%) and *H*. *cymosum* EO (8.24%) was not identified in previous analysis reports of these two essential oils. In this study, γ-Curcumene was found to be present as a prominent constituent (15.76%) in *H*. *odoratissimum* EO, which is much higher than the percentage composition (2.15%) reported in an earlier report [16]; however, ar-curcumene, another dominant compound in this EO (7.63%), has also been reported in the Cameroonian *H*. *odoratissimum* EO in a higher content of 20.3% [19].

**Figure 1:**
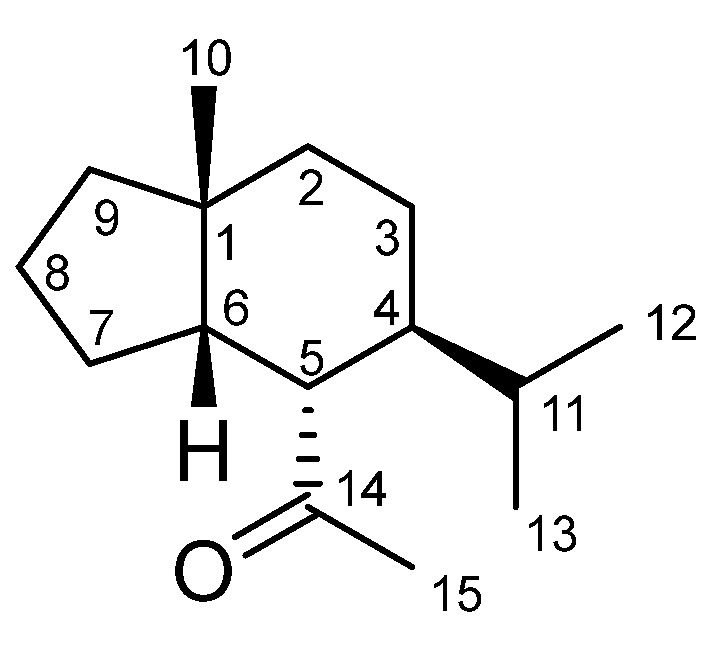
Chemical structure of faurinone.

The major compound in *H*. *petiolare* essential oil (compound **1**; RI = 1568) identified by GC-MS analysis was structurally elucidated as faurinone (Figure 1) through NMR and MS analyses. This sesquiterpene ketone has never been reported before in the essential oil of *H*. *petiolare*. The structural elucidation of compound **2** (RI = 1499) is also discussed further below.

### 2.2. Structural Elucidation of Compound **1**

Compound **1** (5 mg) was identified as faurinone (Figure 1) by MS, ^1^H-NMR, and ^13^C-NMR spectroscopic techniques, and the spectroscopic data were compared with the previously published literature, as presented in the following sections.

#### 2.2.1. Mass Spectrometry

**Figure 2:**
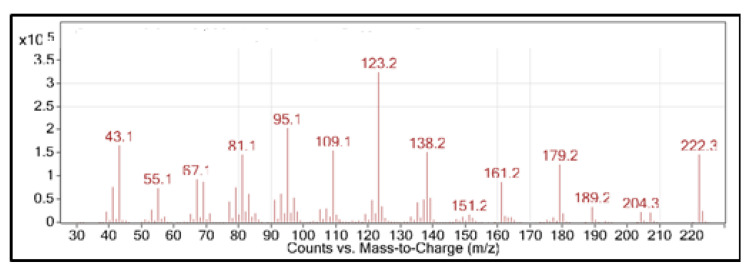
Mass spectrum of faurinone.

The ion fragment peaks obtained in the MS spectrum (Figure 2) were compared to the data first reported by Hikino et al. [25]. The molecular ion [M^+^] at *m*/*z* = 222.3 suggested the molecular formula to be C_15_H_26_O, which translates to the three degrees of unsaturation as it is for faurinone. A base peak was found at *m*/*z* = 123.2, which is typical of isomers of faurinone as described by Weyerstahl et al. [26].

#### 2.2.2. ^1^H NMR Spectroscopy

The ^1^H NMR spectrum showed four methyl signals, and two of them appear at 0.74 ppm and 0.80 ppm (*d*, 6.2 Hz, Me-12, Me-13), and the other two methyl groups appear as singlets at 1.02 (Me-10) and 2.19 (Me-15); the latter is adjacent to a carbonyl group (C-14) (Figure 1). The spectrum also showed a proton resonating at 2.31 (*ddd*, 3.6, 10.7, 14.4 Hz) and assigned to H-4, and another proton at 1.95 (*dd*, 5.0, 10.7 Hz) and assigned to H-5, in addition to a cluster of protons between 1.92 and 0.80 ppm. The proton signals are summarized in Table 3.

#### 2.2.3. ^13^C NMR Spectroscopy

The ^13^C NMR spectrum revealed fifteen carbons that were classified according to DEPT-135 into four methyls, five methylene, four methines, one fully substituted carbon, and a carbonyl group. The peak at δ 212.1 ppm indicated the presence of the carbonyl carbon (C-14) previously reported at δ 211.8 ppm [27]. The remaining peaks from δ 51.9 to 21.4 ppm were representative of typical saturated (sp^3^ hybridized) carbons unaffected by electronegative atoms, as found in the structure of faurinone. Besides the carbonyl carbon, the absent peak at δ 41.6 ppm in the DEPT-135 confirmed the presence of a quaternary carbon at position 1, as reported at the same ppm value by Bos et al. [27] and Weyerstahl et al. [26]. The typical shifts in the saturated carbons in the ^13^C NMR of compound **1** obtained experimentally were in close agreement with the reported values of faurinone [26]. The summary of the comparison with the literature on chemical shifts is presented in Table 4.

### 2.3. Structural Elucidation of Compound **2**

**Figure 3:**
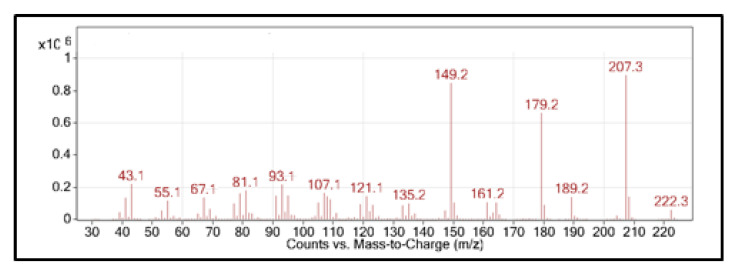
Mass spectrum of compound **2**.

Compound **2** (RI = 1499) could not be fully identified. Weak signals obtained in NMR analysis did not permit complete analysis. However, its mass spectrum showed similarities with the published data. In the literature, compound **2** was identified as β-dihydroagarofuran (RI = 1499) in the essential oil of *H. petiolare* from South Africa as one of the major compounds (19.45–25.65%) by Giovanelli et al. [5]. The mass spectrum of compound **2** (Figure 3) shows similarity to that of β-dihydroagarofuran [22] with the molecular ion [M^+^] = 222.3, the base peak *m*/*z* = 207.3, and other peaks *m*/*z* = 189.2 and, *m*/*z* = 149.2.

### 2.4. Antibacterial Activity: Minimum Inhibitory Concentration (MIC) Using the Broth Microdilution Method

Cutaneous infections pose global health problems [28]. Preventing and treating bacterial skin infections can necessitate topical antimicrobials, which may be an antibiotic. Theoretically, a topical antibiotic presents advantages over systemic administration such as delivering high concentrations of active ingredients to the affected site and less systemic toxicity [29]. The *Helichrysum* EOs were tested against three skin pathogenic bacteria, *S. aureus*, *E. coli*, and *P. aeruginosa*. The MICs of the EOs were taken as the lowest concentration inhibiting visible bacterial growth of strains tested, as detected by the INT (p-iodonitrotetrazolium chloride) reagent and expressed in mg/mL (presented in Table 5).

The MICs of the *Helichrysum* EOs were found between 12.8 and 25.6 mg/mL, whereas the positive control ampicillin exhibited MIC values lower than 0.2 mg/mL (*P*. *aeruginosa* is resistant to ampicillin). Compared to the threshold MIC value of 2 mg/mL for EOs [30], the results indicate that the EOs possess poor antibacterial activities.

### 2.5. Antioxidant Capacities

The magnitude of antioxidative capacities of the *Helichrysum* essential oils was evaluated by four in vitro antioxidant capacity assays: 2,2-diphenyl-1-picrylhydrazyl (DPPH), 2,2′-Azino-bis(3-ethylbenzothiazoline-6-sulfonic acid) (ABTS), ferric reducing antioxidant power (FRAP), and oxygen radical absorbance capacity (ORAC) assays. The results are summarized in Table 6.

In the DPPH assay, the EOs were found to possess a very low percentage of radical scavenging activities (% RSA). The highest % RSA was exhibited by *H*. *petiolare* EO as 14.41 ± 0.51% at 2 mg/mL against 94.94 ± 0.02% for Trolox^®^ positive control at the same concentration. In the ABTS assay, *H*. *petiolare* EO exhibited the highest antioxidant capacity with 84.42 ± 0.43% and 9131.4 ± 45.5 μmol TE/L at 2 mg/mL. The % RSA was close to that of the gallic acid positive control, found as 97.97 ± 0.13% at the same concentration. In the FRAP and ORAC assays, *H*. *odoratissimum* EO was found to exhibit the highest antioxidant capacities at 2 mg/mL with 3026.6 ± 184.6 μmol AAE/L and 6624.8 ± 10.8 μmol TE/L, respectively. In these two assays, the positive controls exhibited 10- and 100-fold higher capacities than the highest-performing essential oil. Additionally, *H*. *petiolare* EO was found inactive in the FRAP assay with an equivalence value of −750.5 ± 11.5 μmol AAE/L, whereas *H*. *cymosum* EO was the second-best performing EO in the assay (897.4 ± 173.1 μmol AAE/L). Overall, using the reference controls as benchmarks, the results suggest that the EOs possess low-to-moderate antioxidant capacities.

### 2.6. Tyrosinase Inhibition

Tyrosinase (EC 1.14.18.1), also known as polyphenol oxidase, is a copper-containing enzyme that has a central role in the production of melanin, the pigment responsible for the color of the skin. It catalyzes the first two steps of the multiphase process of melanogenesis, the biosynthesis of melanin. As tyrosinase inhibitors are increasingly prevalent in cosmeceuticals aiming to reduce hyperpigmentation [31], it is important to investigate EOs as natural options in this regard. In the present work, the selected essential oils were tested in the tyrosinase inhibition assay exploring the monophenolase activity of the enzyme by monitoring the absorbance of L-DOPA (λ_490_ nm) using L-tyrosine as a substrate. The essential oils were tested at 200 μg/mL and 50 μg/mL and compared to kojic acid, a standard tyrosinase inhibitor used in cosmetics, at the same concentrations. The results were obtained, as presented in Table 7.

Overall, the EOs exhibited significantly lower tyrosinase inhibition values than kojic acid at 200 and 50 μg/mL. At both concentrations, the EOs performed near equally in the range of 61.59 ± 10.45 to 63.30 ± 2.35% at 200 μg/mL and 22.22 ± 1.46 to 28.62 ± 0.30% at 50 μg/mL, whereas kojic acid was found as 96.24 ± 3.62% and 98.34 ± 0.80% μg/mL at respective concentrations. Since the enzyme inhibition is concentration dependent, the values obtained indicate that collectively the *Helichrysum* EOs are relatively weak tyrosinase inhibitors.

### 2.7. Sun Protection Factor (SPF)

Solar UV rays are the protagonists in external cutaneous aging in humans and provoke a myriad of dermatological complications including skin cancer [32,33,34,35]. Herein, the SPF values of the *Helichrysum* essential oils were determined by measuring the absorbance of dilute hydroalcoholic solutions of EOs (0.1% *v/v*) at 290–320 nm at 5 nm intervals then calculated using the equation given by Mansur et al. [36]. The results are presented in Table 8.

According to the study, the SPF of the essential oils was found to be 1.511, 0.956, and 0.309, for *H*. *petiolare*, *H*. *cymosum*, and *H*. *odoratissimum* Eos, respectively. As compared to the previously reported threshold SPF value of 2 [38,39], the results may not establish these *Helichrysum* EOs noteworthy for sunscreen formulations.

## 3. Materials and Methods

### 3.1. Plant Material

Two out of the three species studied, *H*. *petiolare* (6.0 kg) and *H*. *cymosum* (3.5 kg), were wildly harvested from the University of the Western Cape campus in December 2018. Their voucher specimens were authenticated by Hlokane Mabela and deposited at the Horticultural Sciences Department of the Cape Peninsula University of Technology. The essential oil of *H*. *odoratissimum* was purchased directly from a local South African establishment (Pure Indigenous [Indigo Trading] African *Helichrysum*, 100% Organic Essential Oil).

### 3.2. Extraction of Essential Oil

The fresh aerial parts (leaves, stems, and flowers) of the plants were subjected to hydrodistillation using the Clevenger-type apparatus for 3 h as per the European Pharmacopeia guidelines [40]. The essential oil was recovered by decantation in glass vials and stored in the dark at 4 °C until further use. The oil yield was expressed as the average percentage of volume in mL per weight in g (% *v/w*) of triplicate analyses.

### 3.3. Gas Chromatography-Mass Spectrometry (GC-MS) Analysis

The GC-MS analyses were carried out according to the procedure previously reported by Kuiate et al. [19] with some adjustments. The instrument consisted of an Agilent GC-7820A fitted with an HP-5MS fused silica column (30 m × 0.25 mm i.d. × 0.25 μm film thickness) and coupled with an Agilent 5977E mass selective compartment (Agilent Technologies, Inc. USA). The oven temperature was programmed at 50 °C for 5 min, 50–220 °C at a rate of 2 °C·min^−1^ then 220 °C temperature hold for 5 min for the first ramp. For the second ramp, the temperature was set to 300 °C at a rate of 25 °C·min^−1^. Helium was used as a carrier gas at 1 mL.min^−1^ flow rate and pressure of 7.6522 psi. Sample injection of 1 μL of 1% (*v/v*) solution diluted in *n*-hexane was splitless and operated at 250 °C. A reference standard of homologous *n*-paraffin series of C_8_-C_20_ (Sigma-Aldrich^®^, Cat no. 04070) was prepared and co-injected under identical experimental conditions as those for samples for the determination of retention indices (RIs). The MS spectra were obtained on electron impact at 70 eV scanning from 30.0 to 650 *m/z*.

The identification of the constituents was achieved by computerized matching (MassHunter software, Agilent Technologies, Inc., Santa Clara, USA) of each mass spectrum generated with those stored in the instrument’s built-in mass spectral libraries (National Institute of Standards and Technologies, NIST), then by comparing the experimental RIs [41] and generated mass spectra with those of the NIST online data collection [24] and the literature [22,23]. The relative amounts of individual constituents were calculated automatically based on the total ion count detected by the GC-MS and expressed as percentage composition.

### 3.4. Isolation and Purification of H. Petiolare Essential Oil’s Components

A silica slurry of 3.547 g of *H*. *petiolare* EO was packed in a silica gel column (40 cm × 4 cm). The separation was performed using a gradient elution of hexane: ethyl acetate (Hex: EA) in order of increasing polarity from 100:0 to 94:6 (Hex: EA). The separation yielded 53 fractions (20–50 mL) labeled as 1–53 which were further concentrated at 45 °C.

Fraction 29 was subjected to preparative thin-layer chromatography (prep TLC) to purify compound **1**. Fraction 29 (20 mg) was dissolved in 750 μL of hexane then 250 μL was loaded on three individual silica gel 60 F254 TLC plates (20 cm × 10 cm; Merck, Germany). Subsequently, the plates were developed at 97:3 hexane: ethyl acetate (double run). Compound **1** was marked under λ_254_ nm, scrapped off, and eluted with hexane. Compound **2** was purified from fraction 31 by prep TLC at 92:8 hexane: ethyl acetate (double run) as previously described.

The MS spectra of compound **1** and compound **2** were obtained by dissolving 0.5 mg in 300 μL of hexane and analyzing the samples by the method previously described. Their ^1^H NMR and ^1^3C NMR spectra were recorded at 20 °C using deuterated chloroform on a Bruker Avance™ 400 MHz spectrometer (Germany). The chemical shifts of ^1^H and ^13^C in ppm (δ) were determined with tetramethylsilane (TMS) used as an internal reference.

### 3.5. Antibacterial Assay

#### 3.5.1. Micro-Organisms

The essential oils were tested against three skin pathogenic bacterial strains. These were one Gram-positive strain, wild-type (WT) *S*. *aureus*, and two Gram-negative strains, wild-type (WT) *E*. *coli* and wild-type (WT) *P*. *aeruginosa*.

#### 3.5.2. Preparation of Media

The bacterial species were resuscitated by inoculation into brain heart infusion (BHI) broth (Oxoid UK, Cat. no. CM1135) and incubated at 37 °C for 24 h, after which, each strain was streaked aseptically onto tryptone soya agar for a single colony formation and incubated at 37 °C for 24 h. The cell suspensions were performed in sterile saline, standardized at 0.5 McFarland standard (Remel™, Kansas, Cat. no. R20410) at 1.5 × 10^8^ colony forming units (CFU)/mL. Then, the working suspensions were obtained by a second 1:100 dilution onto BHI to approximately 106 CFU/mL.

#### 3.5.3. Broth Microdilution Susceptibility Assay

The broth microdilution test was performed, as previously described by Lourens et al. [21] and Sartoratto et al. [42] with slight adjustments. An EO stock solution of 51.2 mg/mL was prepared with a BHI:dimethyl sulphoxide (DMSO) (1:1) solution. In a 96-well plate, 100 μL of BHI was added to the experimental wells in triplicate except in well 1. Then, 200 μL of EO stock solution was added to well 1, from which a serial dilution was performed to the last experimental well. Subsequently, 100 μL of cell suspension was added to establish the two-fold 25.6–0.2 mg/mL sample concentration range and a bacterial cell suspension of approximately 5 × 10^5^ CFU/mL. The plate was incubated at 37 °C for 20 h. After incubation, the antimicrobial activity was detected by adding 40 μL of 0.2 mg/mL INT (Sigma-Aldrich^®^, Cat no. I10406) aqueous solution. The plates were incubated at 37 °C for 1 h. The MICs were defined as the lowest concentration of essential oil that inhibited visible growth, as indicated by the color change of INT. Ampicillin (Sigma-Aldrich^®^, Cat No. A9393) was used as a positive control.

### 3.6. Antioxidant Capacity Assays

#### 3.6.1. 2,2-diphenyl-1-picrylhydrazyl (DPPH) Assay

The DPPH assay was performed according to the method previously described by Bondet et al. [43] with slight modifications. In a clear 96-well plate, 275 μL of DPPH reagent (Sigma-Aldrich^®^, Cat no. D9132) (absorbance of 2.0 ± 0.1 at 517 nm) was added to 25 μL of EO sample and Trolox^®^ (Sigma-Aldrich^®^, Cat no. 238831) positive control (2.0, 1.0, and 0.5 mg/mL). For the blank, ethanol was added instead of the sample. The total volume of the assay was 300 μL. The absorbance was read at 517 nm and 37 °C at the 6 min time point. The EO/Trolox^®^ sample was read in triplicate (n = 3). The % RSA of the samples was calculated using Equation (1).

(1) $${\%\;\mathrm{RSA}}_{6\;\min}=1-\frac{A_{sample}\;}{A_{blank}}$$

where *A_sample_* is the absorbance signal of the EO sample and *A_blank_* is the absorbance signal of the DPPH solution (ethanol in place of the sample) at 517 nm and 6 min. The results were expressed as the mean percentage of triplicate measurements (± standard deviation, SD).

#### 3.6.2. 2,2′-Azino-bis(3-ethylbenzothiazoline-6-sulfonic Acid) (ABTS) Assay

The ABTS assay was performed according to Re et al. [44] with slight modifications. The ABTS radical cation (ABTS•+) (Sigma-Aldrich^®^, Cat no. A1888) stock reagent was produced by reacting 5 mL of freshly prepared 7 mM ABTS solution with 88 μL of a freshly prepared 140 μM K_2_S_2_O_8_ (Merck, Cat no. 105091) then allowing the mixture to sit overnight for 16 h in the dark at room temperature. In a clear 96-well plate, 275 μL of ABTS•+ reagent (absorbance of 2.0 ± 0.1 at 734 nm) was added to 25 μL of each ethanolic Trolox^®^ working standard (50 μM, 100 μM, 150 μM, 250 μM, and 500 μM) and EO sample (2.0, 1.0, and 0.5 mg/mL). Gallic acid (Sigma-Aldrich^®^, Cat No. G7384) was used as a positive control. For the blank, ethanol was added instead of the sample. The total volume of the assay was 300 μL. The absorbance was read at 734 nm and 37 °C at the 6 min time point. The EO sample, working standard, and gallic acid sample were read in triplicate (n = 3). The % RSA of each EO or positive control working solution was calculated using Equation (1), where A_sample_ is the absorbance signal of the EO sample/positive control and A_blank_ is the absorbance signal of the ABTS•+ solution (ethanol in place of the sample) at 734 nm. The results were expressed as the mean percentage of triplicate measurements (± standard deviation, SD). The Trolox^®^ equivalent capacity assay (TEAC) values were reduced from the linear regression (R^2^ = 0.9980) of Trolox^®^ concentrations (μM) and the absorbance readings at 734 nm at 6 min and expressed as mean (±SD) of triplicate measurements in μmol Trolox^®^ equivalents per litre of the sample tested (μmol TE/L).

#### 3.6.3. Oxygen Radical Absorbance Capacity (ORAC) Assay

The ORAC assay was performed according to the method described by Prior et al. [45] with slight modifications. In a black 96-well plate, 12 µL of the Trolox^®^ working solutions (83 µM, 167 µM, 250 µM, 333 µM, and 417 µM were prepared with phosphate buffer at pH 7.4) and EO sample (2.0 mg/mL) were added in triplicate (n = 3). Subsequently, 138 µL of fluorescein solution was added followed by 50 µL of freshly prepared by dissolving 2,2’-Azobis (2-methylpropionamidine) dihydrochloride (AAPH) (Sigma-Aldrich^®^, Cat no. 440914) in phosphate buffer (150 mg of AAPH in 6 mL buffer). (-)-Epigallocatechin gallate (EGCG) (Sigma-Aldrich^®^, Cat no. E4143) was used as a positive control. For the blank, the phosphate buffer was added in place of the sample. The total volume of the assay was 200 µL and the temperature was set at 37 °C. Readings of the EO/EGCG samples (2.0 mg/mL) and Trolox^®^ working standard solutions were taken using the excitation wavelength set at 485 nm and the emission wavelength at 530 nm for 2 h at 1 min reading interval. After analysis, the data points of the blank, EO sample, EGCG sample, and Trolox^®^ working standards were summed up over time to obtain the area under the fluorescence decay curve (AUC). The ORAC values were calculated using the linear regression (R^2^ = 0.9861) equation (Y = aX + c) between Trolox^®^ concentration (Y) (μM) and the net area (blank-corrected) under the fluorescence decay curve (X). The results were expressed as the mean (±SD) of triplicate measurements in μmol of Trolox^®^ equivalents per litre of the sample tested (μmol TE/L).

#### 3.6.4. Ferric Reducing Antioxidant Power (FRAP) Assay

The FRAP assay was conducted as recommended by Benzie and Strain [46] with slight adjustments. Firstly, the fresh blue FRAP reagent was prepared by mixing 30 mL of acetate buffer, 3 mL of 2,4,6-tris [2-pyridyl]-s-triazine (TPTZ) (Merck, Cat no. T1253) with 3 mL of FeCl_3_ solution and 6.6 mL of distilled water. Then, an L-ascorbic acid (Sigma-Aldrich^®^, Cat no. A5960) standard series of 50 μM, 100 μM, 200 μM, 500 μM, and 1000 μM was prepared from a 1 mM of L-ascorbic acid stock solution in distilled water. Lastly, in a clear 96-well plate, 300 μL of the FRAP reagent was added to 10 μL of L-ascorbic acid working standard solutions and EO sample (2.0 mg/mL) in triplicate (n = 3). Gallic acid was used as a positive control. For the blank, the phosphate buffer (pH 3.6) was added instead of the sample. The total volume of the assay was 310 μL. The absorbance of TPTZ-Fe (II) in the samples was read at 593 nm at 37 °C for 30 min. The results were calculated using the linear regression (R^2^ = 0.9965) of the L-ascorbic acid (AA) standard series concentrations (μM) and absorbance signals expressed as mean (±SD) of triplicate measurements in μmol L-ascorbic acid equivalents per litre of the sample tested (μmol AAE/L).

### 3.7. Anti-Tyrosinase Assays

#### 3.7.1. Essential Oil Samples and Positive Control Preparation

EO working solution (10 mg/mL) was prepared with a DMSO: Tween^®^20 (1:1) solution to facilitate dispersion of the oils which was further diluted to 1 mg/mL working solution with methanol. A 10 mg/mL kojic acid working solution was made up of 100% DMSO and then diluted to 1 mg/mL with methanol.

#### 3.7.2. Tyrosinase Inhibition Assays

The tyrosinase inhibition assay was performed as described previously by Popoola et al. [47] and Cui et al. [48] with slight modifications. The concentrations of the EO sample and kojic acid chosen, 200 μg/mL and 50 μg/mL, respectively, were achieved by setting up the 96-well plate in the following order: 70 μL of the sample (1 mg/mL) then 30 μL of tyrosinase enzyme (500 U/mL). Each concentration of the sample and positive control was set up in two different wells whereby, one of the wells received enzyme and the other well had no enzyme volume added. All volume deficits were compensated by adding excess buffer. The negative controls, 10% *v/v* of 1:1 DMSO: Tween^®^20 in methanol for the EO and 10% *v/v* DMSO in methanol for kojic acid were treated the same way. The plate was incubated at 37 °C (±2.0 °C) for 5 min. Thereafter, the reaction was initiated by adding 110 μL of L-tyrosine (2 mM) and subsequently incubated at 37 °C (± 2.0 °C) for 30 min. The absorbance of L-DOPA was read at 490 nm on a Multiskan™ spectrum plate reader (Thermo Fisher Scientific, Waltham, MA, USA). Two independent experiments were carried out in triplicate and the percentage of tyrosinase inhibition was calculated using Equation (2).

(2) $$Tyrosinase\;inhibition\;\left(\%\right)=\frac{\;\left(A-B\right)\;-\;\left(C-D\right)}{\;\left(A-B\right)}\times 100$$

where *A* is the negative control with an enzyme, *B* is the negative control without enzyme, *C* is the EO sample or kojic acid with enzyme and *D* is the EO sample or kojic acid without enzyme. The inhibition percentages were expressed as the mean (± standard deviation) of duplicate measurements. One-way ANOVA was used to compare the absorbance values of the two groups (*p* < 0.05).

### 3.8. Sun Protection Factor (SPF)

The protocol used for this assay was conducted as per Kaur and Saraf [49]. The solubility of the EO in different ratios of ethanol and water was tested by taking 10% to 50% of ethanol in distilled water. The maximum solubility was detected at ethanol: water in a 40:60 ratio, above which turbidity developed. Thereafter, an initial stock solution of 1% *v/v* was prepared by making up 10 µL of the EO to 1 mL of ethanol:water (40:60). Then, out of this stock, 0.1% *v/v* in 40:60 ethanol: water was prepared. Subsequently, 100 µL of the EO aliquot and the blank (ethanol: water, 40:60) were injected into the 96-well plate and read in triplicate (n = 3) over the 290 nm-320 nm range at a 5 nm interval. The SPF value of the essential oil was calculated following the method by Mansur et al. [36]. The mean of the observed absorbance values was multiplied by their respective erythemogenic effect (EE) times solar intensity at wavelength λ values, EE (λ) × I (λ), then their summation was obtained and multiplied with the correction factor (=10). The calculation is described as Equation (3).

(3) $${\mathrm{SPF}}_{\mathrm{spectrophotometric}}=CF\times \overset{320}{\underset{290}{\sum }}EE\;\left(\lambda \right)\times I\;\left(\lambda \right)\times Abs\;\left(\lambda \right)$$

where CF is the correction factor (=10), EE (λ) is the erythemogenic effect of radiation at wavelength λ, I (λ) is the solar intensity at wavelength λ, and Abs (λ) represents the spectrometric absorbance value at wavelength λ. The values of EE (λ) × I (λ) are constant values that were determined by Sayre et al. [37], as shown in Table 9.

## 4. Conclusions

The present work aimed to investigate the chemical composition of three South African *Helichrysum* essential oils and to explore their biological activities in the quest to find medicated fragrant ingredients to be used in cosmetic formulations. The GC-MS and NMR analyses revealed that their major constituents were hydrocarbons and oxygenated monoterpenes (α-pinene, 29.82% in *H. cymosum*; 1,8-cineole, 17.44% in *H. odoratissimum*) and oxygenated sesquiterpenes (faurinone, 20.66% in *H. petiolare*). This is the first report elucidating faurinone in the essential oil of *H. petiolare*. The EOs of all the three reported species of *Helichrysum* in this study had α-pinene and 1,8-cineole in common and as one of the major phytoconstituents. α-pinene and 1,8-cineole are associated with pharmacological activities such as antimicrobial, antioxidant, and antitumor effects. However, the biological evaluation of these EOs did not correspond to the reported pharamacological activities of these phytoconstituents. This investigation reiterates the fact that the bioactive functional property of a natural product such as essential oil cannot be linked to a single compound or a group of compounds; rather, it can be a result of the concerted effect of many secondary metabolites. Among the in vitro biological activities, this study is the first to report tyrosinase inhibition and sun protection factor of these *Helichrysum* essential oils. According to the results obtained, the essential oils possessed low antibacterial, anti-tyrosinase activities and photoprotection but moderately promising antioxidant capacities. This study establishes that *H. petiolare*, *H. odoratissimum*, and *H. cymosum* essential oils have great potential to complement antioxidant formulations.

## Figures and Tables

**Table 1:** Review of previous studies on essential oils of the selected *Helichrysum* species under focus.

Name	Locality	Studies on Essential Oils			References
		Analysis Method	Major Components	Biological Tests	
*Helichrysum petiolare* Hilliard and B.L.Burtt	SA	GC-MS	1,8-Cineole (22.4%), (*E*)-caryophyllene (14.0%), *p*-cymene (9.8%)	Antimicrobial, antioxidant, and anti-inflammatory	Lourens et al. [1]
	SA	GC-MS	Caryophyllenyl alcohol (36.42–45.26%), β-hydroagarofuran (19.45–25.64%), δ-cadinene (3.39–4.76%)	None	Giovanelli et al. [5].
*Helichrysum cymosum* (L.) D.Don subsp. *cymosum*	Tanzania	GC-MS	(*E*)-Caryophyllene (27.02%), caryophyllene oxide (7.65%), *p*-cymene (7.55%).	Antimicrobial	Bougatsos et al. [8].
	SA	GC-MS	1,8-Cineole (20.4%), α-pinene (12.4%), (*E*)-caryophyllene (10.8%)	Antimicrobial and antimalarial and cytotoxic	Van Vuuren et al. [11].
	SA	TLC and GC-MS	1,8-Cineole (20.4–34.6%), (*E*)-caryophyllene (8.4–10.8%), α-pinene (3.6–12.4%).	Antimicrobial	Reddy [12].
	Cameroon	GC-FID and GC-MS	δ-3-Carene (16.1%), (*E*)-caryophyllene (12.0%), camphene (7.4%).	Radical scavenging and antifungal	Tchoumbougnang et al. [13].
	SA	GC-MS	(Z)-β-Ocimene (35.61–50.44%), (*E*)-caryophyllene (15.03–16.62%), α-humulene (5.28–8.68%).	None	Giovanelli et al. [5].
*Helichrysum odoratissimum* (L.) Sweet	Zimbabwe	GC-MS	α-Pinene (15.0%), α-humulene (13.0%), (*E*)-caryophyllene (9.6%).	None	Gundidza and Zwaving [14].
	SA	GC-MS	*p*-Menthone 35.4%, pelugone 34.2%, 1,8-cineole 13.0% (fresh plant material).	None	Asekun et al. [15].
	SA	TLC and GC-MS	(*E*)-Caryophyllene (9.3–25.2%), limonene (11.6–19.6%), and 1,8-cineole (11.2–17.1%).	Antimicrobial	Reddy [12].
	SA	GC-MS	Limonene (14.55%), 1.8-cineole (6.56%), α-pinene (4.20%).	Repellent and fumigation against maize weevil	Odeyemi et al. [16].
	SA	GC-MS	β-Pinene (51.6%), limonene (16.9%), α-humulene (5.6%)	Antimicrobial and cytotoxic	Lawal et al. [17].
	Uganda	GC-MS	Palmitic acid (27.1%), humulene (14.1%), (*E*)-caryophyllene (12.6%).	Antimicrobial	Ocheng et al. [18].
	SA	GC-MS	α-Pinene (4.11–18.39%), (*E*)-caryophyllene (9.67–15.85%), 1,8-cineole (2.74–13.35%).	None	Giovanelli et al. [5].

Additionally, as per the literature, these EOs have not been evaluated before for their antityrosinase activity (Table 1). Therefore, the present research aimed to elucidate the chemical composition of the essential oils of these three selected *Helichrysum* species, and biologically evaluate them for their antimicrobial, antioxidant, antityrosinase, and photoprotective activity.

**Table 2:** GC-MS analysis of the *Helichrysum* essential oils.

Mass Spectral Matching	Composition (%)			Experimental RI	Literature RI	Identification
	*H. petiolare*	*H. odoratissimum*	*H. cymosum*			
α-Pinene	7.49	15.76	29.82	938	939 ^A^	RI, MS
Camphene	-	0.32	0.44	951	950 ^B^	RI, MS
β-Pinene	10.54	5.18	2.56	981	979 ^A^	RI, MS
Myrcene	0.50	0.41	0.78	993	990 ^A^	RI, MS
α-Terpinene	-	1.51	1.83	1017	1017 ^B^	RI, MS
1,8-Cineole	9.87	17.44	15.13	1035	1032 ^B^	RI, MS
(*E*)-β-ocimene	17.21	0.42	8.24	1051	1050 ^A^	RI, MS
β-Ocimene (undefined isomer)	3.79	-	3.26	1057	-	Wb MS
γ-Terpinene	0.73	0.82	2.50	1063	1060 ^B^	RI, MS
allo-Ocimene	6.66	-	3.01	1136	1132 ^A^	RI, MS
Borneol	-	-	0.45	1164	1166 ^B^	RI, MS
Terpinen-4-ol	0.57	0.63	2.18	1176	1177 ^B^	RI, MS
α-Terpineol	-	5.51	0.82	1193	1190 ^B^	RI, MS
Lavandulyl acetate	0.99	-	-	1294	1290 ^A^	RI, MS
Myrtenyl acetate	0.41	-	-	1325	1326 ^A^	RI, MS
α-Copaene	0.65	-	-	1372	1376 ^B^	RI, MS
Unknown	-	1.13	-	-	-	-
Lavandulyl propionate	0.41	-	-	1384	-	Match
Italicene	-	3.24	-	1409	1402 ^B^	RI, MS
(*E*)-Caryophyllene	-	7.30	19.20	14.22	1420 ^B^	RI, MS
α-Humulene	3.01	2.06	0.83	1450	1453 ^B^	RI, MS
Unknown	-	-	0.36	1486	-	-
γ-Curcumene	-	15.76	-	1487	1481 ^B^	RI, MS
Phenyl ethyl 2-methylbutanoate	0.90	-	-	1488	1487 ^A^	RI, MS
Ar-Curcumene	-	7.63	-			
Unknown	5.29	3.06	-	1499	-	-
7-epi-α-Selinene	-	-	0.60	1510	1517 ^B^	RI, MS
Sesquicineole	-	2.75	-	1514	1516 ^A^	RI, MS
Lavandulyl isovalerate	1.28	-	-	1514	1509 ^A^	RI, MS
δ-Cadinene	2.05	1.13	-	1522	1523 ^B^	RI, MS
Unknown	-	0.54	-	1531	-	-
α-Calacorene	0.68	0.40	-	1539	1540 ^B^	RI, MS
Faurinone	20.66	-	-	1568	-	MS, NMR
Caryophyllene oxide	-	1.66	2.65	1578	1580 ^B^	RI, MS
Viridiflorol	-	0.45	-	1585	1591 ^B^	RI, MS
Unknown	0.43	-	-	1602	-	-
Junenol	-	0.59	-	1610	1618 ^A^	RI, MS
Unknown	1.93	-	-	1642	-	-
Unknown	0.62	-	-	1649	-	-
Valeranone	1.07	-	-	1666	1672 ^B^	RI, MS
Monoterpene hydrocarbons:	46.92	24.42	53.24			
Oxygenated monoterpenes:	12.25	23.58	18.58			
Total monoterpenoids:	59.17	48.00	71.82			
Sesquiterpene hydrocarbons:	6.39	37.52	20.63			
Oxygenated sesquiterpenes:	23.01	5.99	2.65			
Total sesquiterpenoids:	29.40	43.51	23.28			
Diterpene hydrocarbons:	0.60	0.72	3.09			
Phenylpropanoids:	0.90	0.00	0.00			
Total identified:	90.07	92.23	98.19			
Unidentified:	8.27	4.73	1.82			
Total	98.34	96.96	100.01			

^A^ = Adams [22]. ^B^ = Babushok et al. [23]. Wb = NIST Chemistry WebBook [24]. MS = In addition to RI, the MS of the analyzed compound matched with the MS of the compound in [22] and/or NIST Chemistry WebBook [24]. Wb MS = The MS of the analyzed compound matched with the compound listed in [24]. Match = no RI or MS available in the literature. The compound was reported solely based on the mass spectral match with NIST14 libraries reported by MassHunter software (Agilent Technologies, Inc., Santa Clara, CA, USA) (Probability < 0.04). Unknown = The MS of the compound could not be matched with the available literature data. U = Undefined. Higher n-paraffin needed.

**Table 3:** Summary of identified protons in the ^1^H spectrum of faurinone.

Title 1	δ (ppm) Multiplicity (*J* = Hz)	
	Experimental	Reported
H-4	2.31 *ddd* (3.6, 10.7, 14.4)	*tt* * (10.4, 3.8) [27]
H-5	1.95 *dd* (10.7, 5.01)	*dd* * (10.4, 4.8) [27]
H-10 (Me)	1.02 *s*	1.03 *s* [25]
H-(12,13) (Me)	0.74 *d* (6.12) 0.80 *d* (6.12)	0.74 *d* (5) 0.81 *d* (5) [25]
H-15 (Me)	2.19 *s*	2.18 *s* [25]

* chemical shifts were not reported.

**Table 4:** Experimental and literature values [27] of ^13^C NMR shifts (ppm) of faurinone.

Carbon *	Multiplicity	Compound 1	Faurinone
CH_2_	*t*	21.38	21.4
C-12	*q*	22.23	22.2
C-13	*q*	23.05	23.0
CH_2_	*t*	26.54	26.5
C-11	*d*	29.04	29.0
C-15	*q*	29.34	29.2
CH_2_	*t*	30.88	30.8
CH_2_	*t*	32.35	32.4
C-10	*q*	36.67	36.6
C-1	*s*	41.60	41.6
C-4	*d*	47.31	47.3
C-5	*d*	49.31	49.3
C-6	*d*	50.94	50.9
C-14	*s*	212.10	211.8

* The methylene groups could not be assigned in this work.

**Table 5:** MICs (mg/mL) of *Helichrysum* EOs and control.

Sample	Micro-Organisms		
	*S. aureus*	*E. coli*	*P. aeruginosa*
*H. petiolare*	>25.6	12.8	12.8
*H. odoratissimum*	12.8	12.8	12.8
*H. cymosum*	>25.6	12.8	12.8
Ampicillin	<0.2	<0.2	R *

* R = resistant.

**Table 6:** Antioxidant capacities of *Helichrysum* EOs in the DPPH, ABTS, FRAP, and ORAC assays.

Sample		DPPH *	ABTS *			FRAP *	ORAC *
	mg/mL	% RSA_6_ min ± SD	% RSA_6_ min ±SD	TEAC (μmol TE/L ± SD)	mg/mL	FRAP (μmol AAE/L ± SD)	ORAC (μmol TE/L± SD)
*H. petiolare*	2	14.41 ± 0.51	84.42 ± 0.43	9131.4 ± 45.5	2	−750.5 ± 11.5	6587.3 ± 126.3
	1	8.98 ± 0.40	77.96 ± 0.71	8445.9 ± 76.1			
	0.5	5.29 ± 0.20	67.08 ±0.76	7281.7 ± 81.5			
*H. odoratissimum*	2	4.09 ± 0.95	60.74 ± 1.24	6603.8 ± 132.6	2	3026.6 ± 184.6	6624.8 ± 10.8
	1	1.27 ± 0.43	46.72 ± 0.96	5103.8 ± 102.7			
	0.5	−0.57 ± 0.03	28.16 ± 0.84	3117.5 ± 89.5			
*H. cymosum*	2	5.58 ± 0.61	40.26 ± 0.33	4412.2 ± 35.7	2	897.4 ± 173.1	6549.7 ± 99.9
	1	3.14 ± 0.00	23.69 ± 0.70	2639.6 ± 75.3			
	0.5	1.58 ± 0.51	10.70 ± 0.22	1250.1 ±23.9			
Trolox^®^	2	94.94 ± 0.02	-	-	-	-	-
	1	94.78 ± 0.06					
	0.5	94.45 ± 0.04					
Gallic acid	2	–	97.97 ± 0.13	605,840 ± 27811.3	2	635,500 ± 4070.9	–
	1		97.96 ± 0.16	355,740 ± 7127.6			
	0.5		98.05 ± 0.03	195,220 ± 6241.5			
EGCG **	–	–	–	–	2	–	26,904 ± 328.2

* Average values of triplicate measurements (n = 3); RSA: radical scavenging activity; SD = standard deviation; RSD = relative standard deviation; TE: Trolox^®^ equivalent; AAE: ascorbic acid equivalent. ** EGCG: (-)-epigallocatechin gallate.

**Table 7:** Summary of the tyrosinase inhibition assay results for the *Helichrysum* EOs at 200 μg/mL and 50 μg/mL.

Samples	Tyrosinase Inhibition (%)	
	at 200 μg/mL	at 50 μg/mL
*H. petiolare*	62.66 ± 11.96	22.22 ± 1.46
*H. odoratissimum*	63.30 ± 2.35	28.62 ± 0.30
*H. cymosum*	61.59 ± 10.45	25.42 ± 1.80
Kojic acid	96.24 ± 3.62	98.34 ± 0.80

**Table 8:** Spectrophotometric absorbances of hydroalcoholic aliquots of the *Helichrysum* essential oils and their calculated SPF.

Wavelength (nm)	EE(λ) × I(λ) ** Employed	Absorbance *		
		*H. petiolare* EO	*H. odoratissimum EO*	*H. cymosum EO*
290	0.0150	0.2999 ± 0.0060	0.0632 ± 0.0020	0.2955 ± 0.0054
295	0.0817	0.2813 ± 0.0079	0.0436 ± 0.0048	0.2244 ± 0.0085
300	0.2874	0.2129 ± 0.0165	0.0354 ± 0.0024	0.1259 ± 0.0063
305	0.3278	0.1290 ± 0.0112	0.0283 ± 0.0011	0.0746 ± 0.0038
310	0.1864	0.0796 ± 0.0070	0.0250 ± 0.0015	0.0478 ± 0.0024
315	0.0837	0.0548 ± 0.0057	0.0235 ± 0.0005	0.0342 ± 0.0015
320	0.0180	0.0384 ± 0.0036	0.0208 ± 0.0010	0.0254 ± 0.0010
Calculated SPF		1.511	0.309	0.956

* Values represent mean absorbance values ± standard deviation of triplicate measurements, n = 3; ** constant values of erythemogenic effect (EE) of radiation with wavelength λ × solar intensity (I) at wavelength λ determined by Sayre et al. [37].

**Table 9:** Relationship between erythemogenic effect and radiation intensity.

Wavelength (nm)	EE × I (Normalized)
290	0.0150
295	0.0817
300	0.2874
305	0.3278
310	0.1864
315	0.0837
320	0.0180
Total	1

## Data Availability

The data supporting reported results can be found at https://etd.cput.ac.za/handle/20.500.11838/3340?mode=simple (accessed on 30 September 2022).
